# Photoperiod and Circadian Regulation in Plants: A Review of Insights from In Vitro Studies

**DOI:** 10.3390/biology14111502

**Published:** 2025-10-27

**Authors:** Adriely Sá Menezes do Nascimento, Juliane Maciel Henschel, Sérgio Heitor Sousa Felipe, Antonia Alice Costa Rodrigues, Fábio Afonso Mazzei Moura de Assis Figueiredo, Tiago Massi Ferraz, Fabrício de Oliveira Reis, Thais Roseli Corrêa, Diego Silva Batista

**Affiliations:** 1 Programa de Pós-Graduação em Ciências Agrárias, Universidade Estadual do Maranhão, São Luís 65055-310, MA, Brazil; adrielysamenezes5@gmail.com (A.S.M.d.N.);; 2 Programa de Pós-Graduação em Agronomia, Universidade Federal da Paraíba, Areia 58397-000, PB, Brazil; 3 Universidade Federal Rural da Amazônia (UFRA), Capitão Poço 68650-000, PA, Brazil

**Keywords:** circadian rhythm, light, tissue culture, physiological plasticity

## Abstract

**Simple Summary:**

Plants rely on internal “biological clocks” to coordinate their growth and development with daily and seasonal changes in light, known as the photoperiod. This review explores how plant tissue culture (growing plants under controlled laboratory conditions) can help scientists better understand how light cycles influence plant rhythms and behavior. By analyzing studies from the scientific literature, the authors show that changes in photoperiod affect not only basic plant processes, such as photosynthesis and metabolism, but also important developmental events like flowering, tuber formation, and growth. The in vitro system allows researchers to study these effects with great precision, helping to reveal how plants adapt to environmental variations. The findings highlight that combining in vitro culture techniques with studies on photoperiod and circadian regulation provides powerful tools to improve plant propagation and increase the production of useful natural compounds. This knowledge can ultimately contribute to the development of more sustainable and resilient agricultural systems in the face of global climate challenges.

**Abstract:**

Plants possess several molecular mechanisms that enable them to adapt their development to environmental changes. Many plant biological processes depend on the circadian rhythm and are regulated by the internal biological clock. Predictable environmental changes, such as variations in photoperiod, can modulate circadian rhythms, allowing organisms to synchronize their biological processes with seasonal conditions. Plant tissue culture is a valuable tool for investigating and monitoring plant plasticity in response to environmental fluctuations, as well as for elucidating the biological changes that occur under these conditions. This review highlights the importance of in vitro culture as a tool to study the physiological plasticity triggered by photoperiod and its interaction with the plant biological clock. To achieve this, a descriptive analysis was conducted through a literature search in the *Scopus* database, followed by a bibliometric analysis to demonstrate the progress in the application of in vitro culture to studies on photoperiod and circadian regulation in plants.

## 1. Introduction

In vitro culture provides sterile propagation of plant cells, tissues, or organs on nutrient media under controlled conditions, where cellular totipotency drives the regeneration of whole plants with defined traits or their plant organs [1,2]. Because of this regenerative potential, in vitro culture underpins research in plant biology, biochemistry, and molecular biology, and supports strategies to overcome agricultural challenges, including those imposed by global climate change [3,4].

Environmental stress factors disrupt biochemical and physiological processes that sustain plant growth and development. However, these stressors also induce phenotypic plasticity through epigenetic mechanisms, which increases tolerance and enhances adaptive responses [5].

Circadian clocks evolved as endogenous ~24 h systems. Environmental cues, particularly those of exceptionally low light intensity, regulate these clocks, enabling organisms to anticipate predictable fluctuations and optimize their adaptation and performance [6,7]. In plants, circadian regulation aligns physiological processes with daily cycles. This alignment drives photosynthesis, carbon metabolism, growth, and flowering, and simultaneously modulates defense pathways and responses to stress [8].

The circadian system relies on interconnected transcriptional–translational feedback loops (TTFLs) that form the central oscillator. Clock genes interact with environmental signals to generate rhythms that control daily patterns of plant behavior [9,10,11,12,13]. Light predominates among these signals because it regulates transcription and translation of rhythmic genes and stabilizes messenger RNA [14]. Temperature also influences central oscillator components, but researchers have not yet fully elucidated the molecular pathways involved [15,16]. The photoperiod, determined by the length of the light phase within the 24 h cycle, governs developmental transitions, including flowering, seed germination, senescence, fruit set, and budburst [17,18,19,20].

When circadian timing fails to align with external rhythms, circadian disruption occurs, resulting in reduced plant growth and developmental performance [10]. This misalignment can affect key physiological processes, such as photosynthesis, hormone signaling, and resource allocation, thereby compromising the plant’s overall fitness. Understanding how circadian regulation mediates stress tolerance is therefore essential for developing cultivars with superior productivity.

Understanding circadian regulation is critical for improving stress tolerance and overall performance in crop cultivars. While in vivo studies provide valuable insights under natural conditions, in vitro culture allows precise control of environmental variables, such as light and temperature, as well as the developmental stage of the explants. This control enables detailed investigation of the interplay between photoperiod, developmental cues, and circadian regulation. This review highlights how in vitro approaches provide a robust framework to study the physiological plasticity of vascular plants at the whole-plant level.

## 2. Circadian Rhythm in Plants

Circadian rhythms (from the Latin circa, “about,” and diem, “day”) coordinate cellular and metabolic processes by anticipating and synchronizing responses in plants and animals [21,22,23]. This rhythmicity enables organisms to adjust essential processes to the most suitable time of day. In plants, circadian rhythms promote growth and development, enhancing fitness when the internal clock is in synchrony with the light–dark cycle [11,24,25].

In general, the plant circadian system is structured into three main pathways that ensure the integration of environmental stimuli and maintain endogenous rhythmicity. The input pathway includes light and temperature receptors responsible for capturing external information. The central oscillator consists of interconnected TTFLs, including the morning and evening loops, which synchronize environmental signals with the expression of clock genes. The output pathway translates this information into physiological and metabolic responses. These components do not act in isolation but rather in an interconnected manner, reinforcing the robustness and plasticity of the system and enabling plants to adjust growth, development, and adaptive responses to dynamic environmental conditions [26,27].

The circadian clock is composed of core genes that interact through multiple TTFLs, in which the transcription of each TTFL accumulates for a defined period within a 24 h cycle, thereby generating rhythmic gene expression [28,29]. However, although the genes are expressed throughout the day, transcriptional phases differ depending on the time of day, driven by the activity of multiple core circadian clock proteins [30].

The circadian clock genes in *Arabidopsis thaliana* are organized into distinct phases of expression that alternate between morning and evening periods. During the morning phase, the main components include CIRCADIAN CLOCK-ASSOCIATED 1 (CCA1), LATE ELONGATED HYPOCOTYL (LHY), REVEILLEs (RVE8, RVE4, RVE6), NIGHT LIGHT-INDUCIBLE AND CLOCK-REGULATED (LNK1, LNK2), and PSEUDO-RESPONSE REGULATORS (PRR9, PRR7) [31,32,33]. In contrast, during the evening phase, the key components are PRR5, PRR3, PRR1 (also known as TIMING OF CAB EXPRESSION 1, TOC1), ZEITLUPE (ZTL), GIGANTEA (GI), LUX ARRHYTHMO/PHYTOCLOCK 1 (LUX/PCL1), and the EARLY FLOWERING genes (ELF3, ELF4) [34,35].

In an integrated manner, most of these proteins participate in light signaling, contributing to the alignment of the circadian clock with environmental cycles. They adjust endogenous rhythmicity daily through the perception of light and temperature cues at dawn and dusk [16,36]. Moreover, these proteins can modify the expression phase of transcripts in response to photoperiod, enabling plants to synchronize their biological processes with seasonal variations [16,37,38]. The circadian clock exhibits a self-sustained oscillation that persists even in the absence of environmental changes, demonstrating its endogenous rhythmicity. This rhythm is entrained by external cues such as light and temperature, allowing plants to synchronize internal processes with the environment. Importantly, the clock also shows temperature compensation, maintaining a consistent period despite fluctuations in ambient temperature. Under environmental stress conditions, plants reorganize their circadian components and activate alternative output pathways, thereby enhancing stress resilience, developmental efficiency, and overall fitness [24,27,39].

In recent years, several studies have identified genes related to the *Arabidopsis* circadian clock in various plant species, primarily those of agricultural interest, including *Pisum sativum*, *Oryza sativa*, *Sorghum bicolor*, *Glycine max*, *Zea mays*, and *Triticum aestivum* [40]. These studies suggest that clock-related genes may contribute to high performance and increased crop productivity. While findings from *Arabidopsis* provide a useful reference, it is important to recognize that the specific roles and regulatory networks of clock genes can differ between species. For example, as observed in *Arabidopsis*, the circadian effect on biomass vigor in hybrids is associated with altered expression of the CCA1 homolog [41]. Similarly, in maize hybrids, CCA1 acts as a morning activator that regulates the expression of photosynthetic genes, promoting photosynthesis and biomass heterosis [42], although the downstream regulatory interactions may not be identical to those in *Arabidopsis*.

In *Arabidopsis*, PSEUDO-RESPONSE REGULATOR (PRR) genes play a central role in the regulation of abscisic acid (ABA) signaling. In *Oryza sativa*, PRR95 acts as a transcriptional regulator rather than a direct transcription factor, modulating the expression of ABA-responsive genes to influence seed germination, seedling growth, and early development [43]. This functional divergence illustrates that PRR genes can have species-specific roles in connecting the circadian clock to hormonal signaling pathways. Comparisons with other species, such as soybean and *Brassica rapa*, further highlight the variability in PRR-mediated regulation of ABA responses, emphasizing the need to consider both conserved and divergent mechanisms when studying circadian–hormone interactions across vascular plants [25,40].

The GIGANTEA (GI) gene was first associated with the effect of late flowering in Arabidopsis [44]. Subsequently, GI was also related to tolerance to stress triggered by several abiotic factors, such as cold [45,46], drought [47,48], and salinity [49,50]. Kim et al. [51] demonstrated that the molecular functions of GI described in *Arabidopsis* are conserved in *Brassica rapa* and suggested that manipulating gene expression can increase tolerance to abiotic stresses, such as salinity, thereby improving crop yield. In *Glycine max*, orthologs of GIGANTEA influence the regulation of flowering time in response to photoperiod and productivity [52].

### Publications on Circadian Rhythm in Plants In Vitro

**Figure 1:**
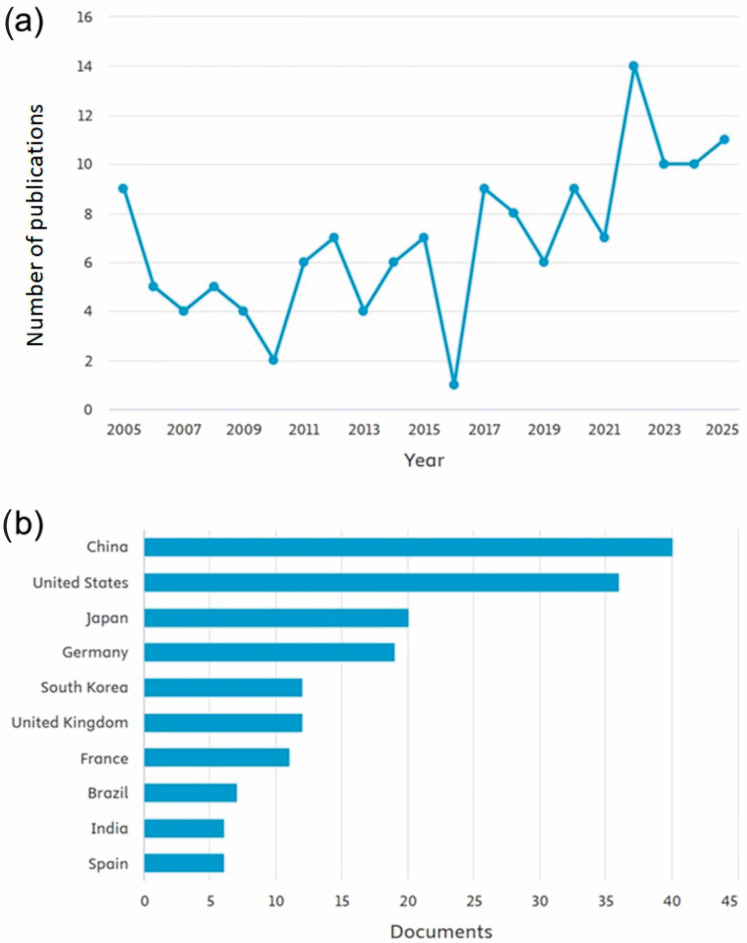
Publication metrics on circadian rhythm in plants in vitro. The search was done with the terms “circadian rhythm”, “plant*”, “in vitro”, filtering only works of the “article” type and in English in the last ten years. Publications over the years (**a**) and countries where most publications occurred (**b**).

Searches in the Scopus database reveal a fluctuating trend in the number of publications containing the terms “circadian rhythm,” “plant*,” and “in vitro” in the title, abstract, or keywords. The search was limited to research papers, excluding review articles, conference papers, or technical notes. In 2016, there was a sharp decline in publications on circadian rhythm in plants under in vitro conditions (only one article). However, this was followed by an increase in 2017 (nine documents). Although the number of publications gradually declined again in 2019, it consistently remained above six per year, reaching a peak in 2022 with 14 publications (Figure 1a). China accounted for the largest number of studies on circadian rhythm in plants in vitro, with approximately 40 publications, followed by the United States with 36 (Figure 1b). These findings highlight the growing global recognition of circadian regulation as a key factor in plant development and productivity. The increase in publications up to 2022 suggests a phase of consolidation in this research area, reflecting both methodological advances in molecular and physiological analyses under controlled conditions and the broader integration of circadian studies into plant biotechnology. The slight decline observed after 2022 may indicate a transition toward more specialized or applied investigations. Overall, these trends point to the need for future studies focusing on diverse plant species, cross-environmental comparisons, and the integration of omics approaches to uncover how circadian regulation can be harnessed to optimize plant growth and resilience.

The circadian clock and photoperiod are intricately interconnected systems that jointly regulate plant development and environmental adaptation. While the circadian clock generates internal rhythms that align physiological and metabolic processes with the day–night cycle, photoperiod provides an external temporal cue that entrains these rhythms to seasonal changes. Through this interaction, plants integrate endogenous timing with external light–dark signals, optimizing processes such as photosynthesis, hormone signaling, and flowering. Understanding this mechanistic connection is essential before exploring how photoperiod specifically influences plant growth and development under in vitro conditions.

## 3. Photoperiod in Plants

The Earth’s rotation and orbit create a predictable daily cycle of light and darkness lasting approximately 24 h. The length of daylight within this cycle, known as the photoperiod, varies with the season and latitude [53,54]. Photoperiod plays a central role in regulating many plants’ biological processes. By conveying information from the internal circadian clock through light perception, plants adjust their growth and development to changing light conditions. This synchronization allows plants to time key developmental events, such as flowering, to occur under optimal seasonal conditions. Importantly, photoperiod-dependent flowering has been shaped by environmental selection: species that flower when days are shortening or lengthening have evolved strategies to maximize reproductive success under their specific climatic conditions, ensuring that reproduction aligns with favorable environmental periods [54,55,56,57].

The regulation of flowering by day length is a well-established concept in plant physiology. Since the pioneering work of Garner and Allard [58], who first demonstrated that photoperiod rather than photosynthate availability determines flowering time, subsequent studies have built upon this foundational discovery to elucidate its molecular basis [58,59,60,61,62]. Based on their photoperiodic responses, plants are generally classified into three categories: long-day plants, which flower under long-day (LD) conditions; short-day plants, which flower under short-day (SD) conditions; and day-neutral plants, which show little or no sensitivity to photoperiod and tend to maintain stable developmental processes regardless of day length [63,64,65].

Studies using light pulses to interrupt the night period have shown that plants, regardless of their previous classification, respond to the period of darkness, not specifically to day length [66,67]. More recent research shows that photoperiod-dependent flowering is, in fact, a response driven by the circadian clock. Nevertheless, the terms mentioned remain useful, as they categorize plants according to their photoperiodic response [59,68].

### Publications on Photoperiod in In Vitro Plants

**Figure 2:**
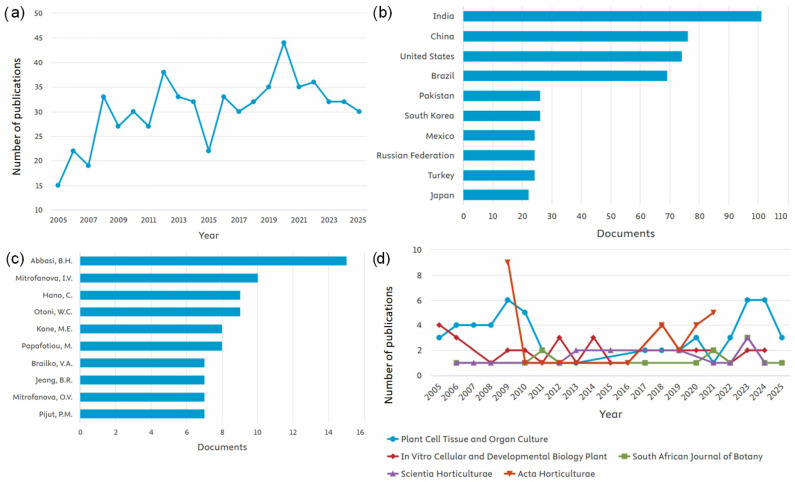
Publication metrics on photoperiod in plants in vitro. The search was done with the terms “photoperiod”, “plant*”, “in vitro”, filtering only works of the “article” type and in English in the last ten years. Publications over the years (**a**), countries where most publications occurred (**b**), authors who have published the most on the topic (**c**) and journals that published the most on the topic (**d**).

Searches in the *Scopus* database show fluctuations in the number of publications containing the terms “photoperiod,” “plant*,” and “in vitro” in the title, abstract, or keywords. The search was limited to research papers, excluding review articles, conference papers, or technical notes. Between 2005 (15 documents) and 2025 (30 documents), the number of studies addressing photoperiod in plants under in vitro conditions increased. Despite oscillations over the 20-year period, 2020 marked a peak with 45 publications (Figure 2a). India contributed the highest number of publications on this topic, with 100 documents, followed by China (74) and the United States (73) (Figure 2b). This predominance of India and China likely reflects their strong investment in plant biotechnology and tissue culture research, as well as the high diversity of plant species cultivated and studied under controlled conditions in these countries. Notably, the most prolific author was Abbasi, Bilal Haider, from Pakistan (Figure 2c), further highlighting the significant contribution of Asian research groups to this field. The journal with the greatest number of recent publications was Plant Cell, Tissue and Organ Culture (57 articles), followed by In Vitro Cellular and Developmental Biology—Plant (37) and Acta Horticulturae (28) (Figure 2d). These numbers highlight the importance of in vitro culture as a tool for investigating photoperiod in plants.

Over the last four years, the number of publications focusing exclusively on photoperiod has declined, likely because many fundamental mechanisms have already been characterized. However, photoperiodic studies increasingly address complex interactions with other factors, such as temperature, light intensity, plant growth regulators, and elicitors, which often require whole-plant experiments to capture relevant phenotypes, including photoperiod-dependent flowering. In vitro approaches provide an alternative framework to study these interactions under controlled conditions, allowing precise manipulation of environmental cues. It is possible that recent publications investigating these multifactorial interactions are not fully captured in conventional literature searches, suggesting that alternative search strategies may be required to comprehensively assess the current research landscape.

Most publications on photoperiod under in vitro conditions investigated its interactions with plant growth regulators (PGRs) (Table 1). Many of these studies described the development of efficient micropropagation protocols, focusing on promoting plant growth and development (primary metabolism) [69,70,71], as well as enhancing the production of bioactive compounds (secondary metabolism) [71,72,73]. However, the bibliometric data reveal notable knowledge gaps. Few studies have explored how photoperiod influences gene expression networks and signaling pathways under in vitro conditions, or how these responses compare with those observed in whole plants grown ex vitro. Additionally, the limited number of studies on non-model or economically important species indicates an opportunity to expand research beyond traditional model systems. Addressing these gaps would contribute to a more comprehensive understanding of how photoperiod modulates physiological plasticity in controlled environments.

## 4. Response of In Vitro Grown Plants to Photoperiod and Variation in Circadian Rhythms

In vitro culture has a practical purpose, as it enables large-scale plant propagation. This direct application consolidates it as an essential tool that also facilitates biotechnological applications in plant breeding, particularly in stages involving genetic transformation [1,4]. Moreover, in vitro culture is widely employed in studies of plant biology, biochemistry, and molecular biology, as it allows precise control of growth conditions (light intensity and quality, photoperiod, temperature, among others) and accelerates data generation by reducing the time required for treatments to express their phenotypes [4].

In this context, researchers have investigated photoperiodic rhythmicity and other environmental conditions using in vitro culture as an effective tool, since the system enhances growth and yield while conferring high tolerance to environmental stresses [88,92,101]. Because tissue culture provides a controlled, uniform, and efficient environment, it becomes the method of choice in studies that require strict control and elimination of external variability—conditions difficult to achieve with conventional approaches [4]. For this reason, Takase et al. [81] reported that when attempting to control day length in *Gentiana triflora* under field conditions, they opted to use in vitro-grown plantlets.

Despite its advantages, in vitro culture has inherent limitations. Artificial light sources often differ in spectral quality and intensity from natural sunlight, potentially affecting photoperiodic responses. Environmental conditions such as humidity, CO_2_ concentration, and nutrient availability are tightly controlled in vitro but differ significantly from field environments, which can influence plant physiology and development [95,101]. Consequently, photoperiodic or stress responses observed in vitro may not always translate directly to ex vitro or field conditions. Researchers must carefully consider these factors when extrapolating findings, and complementary studies under natural conditions are often necessary to validate in vitro observations.

### 4.1. Plant Flowering Time Responses to Photoperiod

Several studies have demonstrated how plants respond to photoperiod under in vitro conditions, with one of the most relevant effects being the regulation of flowering time. In many species, the expression of the FLOWERING LOCUS T (FT) gene results in the production of a systemic signaling molecule that acts as a trigger for the onset of flowering. This molecule is synthesized in the leaves and transported to the shoot apical meristem, where it induces flower formation [109].

The photoperiod-dependent flowering mechanism induced by FT expression is best characterized in long-day plants. In *Arabidopsis thaliana*, long-day conditions induce high levels of FT expression, which accelerate flowering, whereas short-day conditions lead to very low FT expression [110]. The induction of FT depends on both day length and the transcriptional activator CONSTANS (CO), whose expression is tightly regulated by the circadian clock [111]. According to the external coincidence model, the accumulation of CO transcripts occurs from late afternoon to evening, under circadian control, coinciding with light exposure, particularly during summer [112]. Under these conditions, the CO protein is stabilized, leading to increased FT expression and the promotion of flowering. Thus, the flowering response represents a clear example of how the molecular components of the circadian clock (such as CO and FT) integrate environmental light cues to generate coordinated developmental outcomes. This connection between clock-regulated gene expression and phenotypic flowering behavior illustrates the close interplay between molecular timing mechanisms and photoperiodic adaptation in plants.

In *Cannabis sativa* grown in vitro, flowering induction depends not only on photoperiod but also on the physiological state of the explants prior to exposure to inductive conditions. In the study by Mahlberg and Hemphill [21], flowering was observed when in vitro-grown plantlets, already established with 6–8 fully expanded leaves, were exposed to photoperiods of 13.2 h or less per day, or, more precisely, to dark periods of at least 10.8 uninterrupted hours. Similar results were reported by Potter [88], who found that low light intensity during the floral induction stage optimized flower formation, emphasizing that induction efficiency depends on the developmental maturity of the cultured plantlets.

In *Plumbago auriculata*, both temperature and photoperiod influenced flowering. In vitro plantlets with 5–7 expanded leaves showed inhibited floral bud development under long photoperiods (20 h), whereas an intermediate photoperiod (16 h) promoted rapid floral induction and the formation of complete, morphologically normal floral structures [89]. These findings highlight the importance of considering the plant’s developmental stage before induction when evaluating photoperiodic responses under in vitro conditions.

A study on *Bletia urbana*, an endangered orchid species, showed that a neutral photoperiod (12 h) resulted in the highest flowering rate [102]. This outcome may be associated with adaptations to its natural tropical habitat, where day length and night length show little seasonal variation.

### 4.2. Responses on Primary Metabolism

The control of photoperiod, together with circadian rhythm variation, is also an important tool explored in in vitro culture to promote plant growth and development (primary metabolism). Zhu et al. [74], studying the relationship between putative lunularic acid (AL) levels and growth variation in *Marchantia polymorpha* according to the circadian clock, observed that shoot growth rates were generally higher during the light period than during the dark period. This was accompanied by changes in endogenous AL concentrations, indicating that AL synthesis is closely related to light intensity.

Plant growth and development follow a rhythmic day–night pattern regulated by the circadian clock and light-responsive genes. Key circadian clock components, such as *CCA1*, *PSEUDO-RESPONSE REGULATOR* (*PRR*) genes, and *GIGANTEA* (*GI*), coordinate the timing of gene expression and metabolic activity [6,8]. In *Gossypium hirsutum*, small peptides regulated by the circadian clock, such as *GhRALF1*, may exert rhythmic inhibitory effects on fiber elongation, likely acting downstream of these clock components to synchronize fiber growth with the daily light–dark cycle [76]. By linking clock gene activity to fiber cell metabolism, these observations highlight how circadian regulation underpins primary growth processes in cotton.

In *Solanum tuberosum*, tuberization and tuber yield are strongly affected by day length. A photoperiod of 8 h combined with 3.8 g L^−1^ KNO_3_ resulted in the earliest onset of microtuber formation, at 8 days after explant planting (DAP) [97]. Under a 16 h photoperiod with 1.9 g L^−1^ KNO_3_, tuberization occurred only after 11 DAP; however, this condition increased the number of microtubers by 30% compared with the 8 h photoperiod [97]. In contrast, another study reported that darkness induced microtubers in a shorter time (8.85 days) and reached the highest tuberization percentage (80%). On the other hand, the greatest number of microtubers per explant and the maximum mean fresh weight (207 mg) were obtained under short-day conditions (8 h light) [98].

Tuberization in *Solanum tuberosum* is a short-day–induced process and involves a regulatory mechanism analogous to the flowering control in *Arabidopsis thaliana*. Under long-day conditions, circadian clock–regulated genes promote the degradation of the *Solanum CYCLING DOF FACTOR 1* (*StCDF1*), a transcriptional repressor of the *CONSTANS*-like gene *StCOL1*. When *StCDF1* is degraded, *StCOL1* becomes active and induces the expression of *StSP5G*, which acts as a mobile repressor of tuberization by inhibiting the *StSP6A* gene, a tuberigen homologous to *FT* [113]. Conversely, under short-day conditions, *StCDF1* remains stable and represses *StCOL1*, leading to reduced *StSP5G* expression and allowing *StSP6A* to accumulate, which promotes tuber formation. This finely tuned regulatory cascade illustrates how photoperiodic signals, integrated through circadian clock components, control developmental phase transitions in potato in a manner similar to the flowering pathway in *Arabidopsis*.

Photoperiod also influences seed germination under in vitro conditions. In African *Aloe* species (*A. modesta*, *A. peglerae*, *A. reitzii*), germination speed decreased under long photoperiods (16 h and 24 h) [84]. Similarly, in *Anacamptis longicornu* and *Ophrys panormitana*, germination occurred only under a neutral photoperiod (12 h, 18 ± 1 °C), whereas no germination was observed under 16 h light at 25 ± 1 °C [106]. By contrast, in *Origanum dictamnus*, the highest germination rates up to day 12 of the experiment were obtained under a 16 h photoperiod [103]. In two-year-old scarified seeds of *Anthyllis barba-jovis*, germination ranged from 89 to 97% at 15 and 20 °C under a 16 h photoperiod but dropped to ~84% under continuous darkness [80].

In *Cunninghamia lanceolata* seedlings cultured in vitro under three photoperiods (8, 16, and 24 h), the best development occurred under a 16 h photoperiod, which promoted higher rooting rates, root length, and root volume [91]. In contrast, in *Cydonia oblonga* shoots, the greatest number and longest roots were observed in shoots exposed to 10 days of continuous darkness [78].

Biomass accumulation is also strongly influenced by photoperiod. In callus cultures of *Moringa oleifera*, the highest biomass accumulation was observed under continuous white light (24 h), whereas the lowest occurred under continuous darkness [71]. A similar trend was reported for *Lippia alba* [95] and *Rheum rhaponticum* ‘Raspberry’ [73], where longer photoperiods (16 and 24 h) increased dry mass, while shorter photoperiods (4 h and 10 h) reduced it. In *Artemisia tilesii*, however, hairy roots accumulated more biomass in darkness, but these roots were white, elongated, and less branched [85].

Leaf area can also be influenced by photoperiod. In *Pfaffia glomerata*, leaf area increased under continuous light (24 h) [101]. A similar response was observed in *L. alba* seedlings [95], which developed larger leaves with increasing photoperiod up to 24 h, while shorter photoperiods (4 and 8 h) resulted in smaller leaf areas. In these cases, sucrose supplementation in the medium may have positively contributed to this response under continuous light. In contrast, in *Alocasia amazonica*, larger leaf areas were obtained under shorter photoperiods (8/16 h L/D, 12/12 h L/D) [114].

A reduction in leaf area under short photoperiods may reflect lower photosynthetic performance. In *L. alba*, a shorter photoperiod (4 h) led to reduced concentrations of photosynthetic and photoprotective pigments (carotenoids) [95]. Similar reductions were observed in *P. glomerata* [101] and in *C. lanceolata* under an 8 h photoperiod [91].

### 4.3. Responses on Secondary Metabolism

Several studies confirm that photoperiod exerts a strong influence on the production of secondary metabolites. Although most investigations have been conducted under ex vitro conditions, in vitro studies are increasing due to the possibility of tighter environmental control and the application of elicitors to specific metabolic pathways [115].

In *Lippia alba*, photoperiod modulated the essential oil profile: despite no changes in the relative expression of circadian clock genes, plants grown under continuous light (24 h) showed a significant increase in linalool content compared with 8 h and 16 h photoperiods [95]. Similar results were reported in callus cultures of *Kaempferia galanga*, where a 12/12 h light/dark regime enhanced the accumulation of flavonoids and terpenoids, while production declined sharply under continuous darkness [99]. Likewise, *Basella rubra* cultures accumulated higher levels of phenolics under a 16 h photoperiod than under continuous light or continuous darkness [93].

In *Artemisia ludoviciana*, a 16 h light photoperiod not only enhanced achillin synthesis but also increased callus induction compared with complete darkness [83]. In callus cultures of *Moringa oleifera*, continuous white light (24 h) resulted in the highest accumulation of phenolics (TPC: 18 mg g^−1^), phenolic production (TPP: 287 mg L^−1^), and flavonoids (TFC: 15 mg g^−1^; TFP: 212 mg L^−1^), while also positively regulating the transcription of flavonoid biosynthetic genes [71].

In vitro culture has also proven valuable for understanding the implications of photoperiod in circadian rhythms. In *Solanum lycopersicum*, the interaction between photoperiod and circadian oscillations modulated the phase of cyclic gene expression, improving crop adaptation to different day lengths in latitudes outside the tropics [116]. In *Stevia rebaudiana*, a short-day species with a critical photoperiod of 12–13 h, a 12/12 h regime promoted reproductive development, whereas 15/9 h and 16/8 h photoperiods favored vegetative growth. Conversely, photoperiods shorter than 12 h triggered premature and undesirable reproductive development, leading to reduced steviol glycoside (SG) content [117].

## 5. Conclusions

This review demonstrates that in vitro systems provide a robust framework for studying circadian regulation in vascular plants, enabling precise analysis of how internal biological rhythms coordinate physiological and developmental processes. By offering tightly controlled experimental conditions, in vitro cultivation allows researchers to dissect interactions between the circadian clock and environmental cues such as photoperiod, light intensity, and temperature. The precision and reproducibility of this approach strengthen its potential for revealing the mechanisms underlying physiological plasticity and stress adaptation.

Within this framework, photoperiod has been shown to directly modulate key processes including primary and secondary metabolism, flowering, tuberization, and biomass accumulation. These findings confirm that in vitro cultivation uncovers adaptive mechanisms that may be difficult to observe under ex vitro conditions. Therefore, the integration of circadian studies and in vitro cultivation represents a powerful strategy to advance both plant propagation and the production of valuable secondary metabolites. This research field offers important opportunities for applying biotechnology to address climate change challenges and promote more sustainable agricultural systems.

## Figures and Tables

**Table 1:** Summary of the main studies focused on plant responses to circadian rhythms and/or photoperiod under in vitro cultivation.

Species	Propagation Method	Effects Studied	Response Type	Reference
*Suaeda glauca*	cotyledons, hypocotyls, and leaves	Explant type, plant growth regulators, and photoperiod	Molecular mechanisms and primary metabolism	Mohammadi et al. [69]
*Marchantia polymorpha*	buds	Light intensity	Molecular mechanisms and primary metabolism	Zhu et al. [74]
*Schisandra chinensis*	embryos	Light intensity	Primary metabolism	Sun et al. [75]
*Gossypium hirsutum*	ovules	Photoperiod	Primary metabolism	Wang et al. [76]
*Cocos nucifera*	embryos	CO_2_ concentrations, light intensities, light qualities, and photoperiod	Primary metabolism	Mu et al. [77]
*Sequoia sempervirens*	shoots	Culture medium and photoperiod	Secondary metabolism	Kirakosyan et al. [72]
*Anthyllis hermanniae*	shoots	Plant growth regulators and photoperiod	Primary metabolism	Martini et al. [70]
*Moringa oleifera*	callus	Light quality and photoperiod	Primary and secondary metabolism	Bajwa et al. [71]
*Cydonia oblonga*	shoots	Plant growth regulators and photoperiod	Primary metabolism	Durul et al. [78]
*Rheum rhaponticum*	shoots	Temperature and photoperiod	Primary and secondary metabolism	Wojtania et al. [73]
*Solanum tuberosum*	nodal segments	Gene expression	Molecular mechanisms and primary metabolism	Wang et al. [79]
*Anthyllis barba-jovis*	seeds	Temperature and photoperiod	Primary metabolism	Bertsouklis et al. [80]
*Gentiana triflora*	shoots	Gene expression	Molecular mechanisms	Takase et al. [81]
*Spiranthes ochroleuca*	seeds	Culture medium and photoperiod	Primary metabolism (germination)	Zale et al. [82]
*Artemisia ludoviciana*	nodal segments	Plant growth regulators and photoperiod	Primary and secondary metabolism	Ramos et al. [83]
*Aloe sul-africana*	seeds	Temperature and photoperiod	Primary metabolism (germination)	Amoo et al. [84]
*Artemisia tilesii*	roots	Phenylalanine concentrations and light	Primary and secondary metabolism	Bohdanovych et al. [85]
*Paphiopedilum* spp.	shoots	Plant growth regulators and photoperiod	Molecular mechanisms and primary metabolism	Istiqomah et al. [86]
*Artemisia alba*	shoots	Plant growth regulators and photoperiod	Primary and secondary metabolism	Pecheva et al. [87]
*Cannabis sativa*	stem segments	Culture medium and photoperiod	Flowering	Lavie et al. [88]
*Plumbago auriculata*	shoots	Temperature and photoperiod	Flowering	Shen et al. [89]
*Curcuma caesia*	shoots	Plant growth regulators and photoperiod	Primary metabolism	Sarma et al. [90]
*Cannabis sativa*	shoots	Photoperiod	Primary metabolism and flowering	Moher et al. [18]
*Cunninghamia lanceolata*	shoots	Photoperiod and light quality	Primary metabolism	Xu et al. [91]
*Narcissus tazzeta*	bulbs	Plant growth regulators and photoperiod	Primary and secondary metabolism	Rahimi et al. [92]
*Basella rubra*	callus	Plant growth regulators and photoperiod	Primary and secondary metabolism	Kumar et al. [93]
*Triticum aestivum*	seeds	Culture medium and photoperiod	Primary and secondary metabolism	Virdi et al. [94]
*Lippia alba*	nodal segments	Photoperiod	Primary and secondary metabolism	Castro et al. [95]
*Solanum tuberosum*	shoots	Photoperiod and silver thiosulfate concentrations	Primary metabolism	El-Sayed et al. [96]
*Solanum tuberosum*	stem segments	Photoperiod and KNO_3_ concentrations	Primary metabolism	Choirunnisa & Wardana. [97]
*Solanum tuberosum*	shoots	Culture medium and photoperiod	Primary metabolism	Wafa et al. [98]
*Kaempferia galanga*	rhizome shoots	Plant growth regulators and photoperiod	Primary and secondary metabolism	Shofiyani et al. [99]
*Ficus palmata*	axillary shoots	Culture medium and photoperiod	Primary metabolism	Ahmed et al. [100]
*Pfaffia glomerata*	nodal segments	Photoperiod	Primary and secondary metabolism	Fortini et al. [101]
*Bletia urbana*	protocorms	Plant growth regulators and photoperiod	Primary metabolism and flowering	Rodríguez et al. [102]
*Origanum dictamnus*	seeds	Plant growth regulators and photoperiod	Primary metabolism (germination)	Sarropoulou et al. [103]
*Vaccinium floribundum*	seeds and shoots	Medium, photoperiod, and temperature	Primary metabolism	Meneses et al. [104]
*Fagonia indica*	stem explants	UV-C regimes and photoperiod	Primary and secondary metabolism	Abbasi et al. [105]
*Anacamptis longicornu* e *Ophrys panormitana*	seeds	Culture medium, temperature, and photoperiod	Primary metabolism (germination)	Arcidiacono et al. [106]
*Solanum tuberosum*	cuttings	Gene expression, culture medium, and photoperiod	Molecular mechanisms and primary metabolism	Kondhare et al. [107]
*Ligaria cuneifolia*	embryos	Plant growth regulators and photoperiod	Primary and secondary metabolism	Ricco et al. [108]

## Data Availability

Data will be made available on reasonable request to the corresponding authors.
